# Regional nerve block in postoperative analgesia after cesarean section: A narrative review

**DOI:** 10.1097/MD.0000000000041159

**Published:** 2024-12-27

**Authors:** Yongyi Qin, Yujiao Yang, Sulan Qin, Zhaohui Xiong

**Affiliations:** a Clinical School of North Sichuan Medical College, Nanchong, Sichuan, China; b Department of Anesthesiology, Guang’an People’s Hospital, Guang’an, Sichuan, China.

**Keywords:** cesarean section, postoperative analgesia, regional block

## Abstract

Of all obstetric operations, cesarean section is one of the most common. The impact of postoperative pain on physical and mental health in women cannot be ignored. Moreover, effective postoperative analgesia is essential in women who have given birth. Traditional systemic analgesic methods (intravenous analgesia, oral analgesics, etc) are often accompanied by adverse reactions that are positively correlated with the drug dosage. Regional nerve block is an analgesic and anesthetic technique that temporarily blocks nerve conduction by injecting local anesthetics around the nerve roots, nerve trunks, nerve plexus, ganglia, or surgical area, thereby alleviating or eliminating pain. Currently, the regional block techniques used for postoperative analgesia following cesarean section include paravertebral nerve block, transversus abdominis plane block, rectus sheath block, quadratus lumborum block, ilioinguinal-iliohypogastric nerve block, erector spinae block, wound infiltration analgesia, and intraperitoneal infusion of local anesthetics. These regional block techniques hold great promise for providing effective postoperative analgesia after cesarean section, each with unique advantages. Moreover, regional blocks have a unique place in multimodal analgesia protocols following cesarean section and are increasingly used in clinical practice for analgesia after cesarean section. This review provides an overview of the regional nerve block techniques used for postoperative analgesia following cesarean section, discusses their benefits and drawbacks, and provides a reference for choosing postoperative pain management following cesarean delivery, offering a hopeful outlook for improved patient care.

## 1. Introduction

For most women who undergo cesarean delivery, it is fundamental to achieve better outcomes and optimal recovery, in which pain is effectively controlled. A previous study revealed that the occurrence of moderate-to-severe acute pain 24 hours after cesarean section was 21.0%.^[1]^ Severe acute postpartum pain can lead to chronic pain. In another study, in contrast to women experiencing minor postpartum pain, women with severe acute postpartum pain were 2.5 times as likely to suffer from persistent pain and 3.0 times as likely to suffer from postpartum depression.^[2]^ Ultrasound-guided regional nerve block techniques have become a growing field in obstetric anesthesiology. These regional block analgesics have been incorporated into modern obstetric anesthesia and analgesia clinical practice to help parturients minimize pain after cesarean section and reduce the dosage and side effects of opioids.^[3]^ This article reviews commonly used regional nerve block analgesia methods after cesarean section and their advantages and disadvantages. It also aimed to provide a reference for choosing postoperative pain management following cesarean delivery.

## 2. Material and Methods

The China National Knowledge Infrastructure (CNKI), PubMed, and Google Scholar databases were searched for literature up to March 2024. The search terms were “postoperative analgesia after cesarean section,” “regional block,” “nerve block,” “paravertebral nerve block,” “transversus abdominis plane block,” “ilioinguinal-iliohypogastric nerve block,” “rectus abdominis sheath block,” “lumbar-square muscle block,” “erector spinae muscle block,” “wound-infiltration analgesia,” and “intraperitoneal instillation of local anesthetics.” Relevant literature was screened by reading. The time frame of the literature search was narrowed from 2001 to 2023. The inclusion criteria were reviews, randomized controlled trials, systematic reviews, meta-analyses, case reports, and monographs related to nerve blocks, regional blocks, and post-cesarean section analgesia. The exclusion criteria were literature not associated with the study, duplicated or controversial literature, animal-based trials, trial work for preclinical, and conference abstracts. The search was conducted by 2 researchers who reviewed the sources and content of the literature. Seventy-one papers were included in the analysis.

### 2.1. Paravertebral nerve block

Paravertebral nerve blocks are extensively used for analgesia during abdominal and thoracic surgeries. The spinal nerve roots are located in the paravertebral space after exiting the epidural space. It is an area composed of the posterior segment of the superior costo-transverse ligament, anterior aspect of the pleura, and medial aspect of the vertebral body. The sympathetic trunk was located near this space.^[4]^

For a cesarean section with a Pfannenstiel skin incision, a T_12_-L_1_ paravertebral nerve block is usually performed bilaterally.^[5]^ Although the T_12_-L_1_ paravertebral block may not completely cover the L_1_-innervated Pfannenstiel incision, it may diffuse into the epidural space through the medial intervertebral foramen, reducing visceral pain after cesarean delivery by blocking sympathetic nerves. It has unique advantages over the abdominal wall block, which only targets the abdominal cutaneous nerve.^[6]^ Typical drugs provide analgesia for 9 to 12 hours and may require readministration if the catheter is not placed.^[7]^

Compared with epidural analgesia, paravertebral block has less of an effect on lower limb muscle strength and fewer adverse symptoms such as hypotension and urine retention.^[8]^ The injection sites and efficacy of the paraspinal nerve block can be accurately assessed with ultrasound because of its wide use. Ultrasound guidance allows real-time visualization of the needle and surrounding structures, ensuring precise placement and reducing the risk of complications. Ultrasonography can clearly distinguish the paraspinal block from the erector spinae plane block. Although few studies have compared anatomical landmark-guided and ultrasound-guided paravertebral nerve blocks, ultrasound-guided paravertebral nerve blocks have better analgesic effects and a higher success rate after surgery.^[9,10]^ Nonetheless, there is little evidence regarding paravertebral nerve blocks for pain after cesarean delivery, and there is a lack of randomized controlled trials (RCTs) and evidence beyond case reports. This highlights the need for further research and clinical trials to fully understand the potential of paravertebral nerve blocks for postoperative analgesia after cesarean delivery.

### 2.2. Transversus abdominis plane block

The muscular layer of the anterolateral abdominal wall comprises the rectus abdominis, internal oblique, external oblique, and transversus abdominis. The transversus abdominis plane, which is located between the transversus abdominis and internal oblique, is in the thoracolumbar nerve T_6_-L_1_.^[11]^ Transversus abdominis plane (TAP) blocks are the most researched trunk nerve blocks used during cesarean sections. Ultrasound-guided punctures, including the lateral, posterior, and anterior subcostal approaches, accurately locate the needle and have replaced the superficial anatomical landmark guidance.^[12]^ In recent years, TAP block has been widely utilized for the treatment of postoperative pain following cesarean delivery, which can effectively block the abdominal wall nerve and relieve incision pain in parturients after cesarean section; however, it has a poor effect on visceral or spasmodic pain, such as uterine contraction.^[13]^

One study showed that the posterior approach reduced opioid use 36 hours after cesarean delivery and pain scores 24 hours after surgery.^[14]^ A meta-analysis comparing the duration of lateral and posterior TAP blocks revealed similar findings.^[15]^ Few studies have investigated the differences in the analgesic effects of the posterior and lateral TAP block approaches. Moreover, evidence suggests that the ultrasound-guided posterior approach is the most effective technique for postoperative analgesia after cesarean delivery.

Two meta-analyses revealed a decrease in pain ratings while at rest with vs without TAP block.^[16,17]^ However, studies have shown inconsistent pain reduction with TAP block, which, without intrathecal morphine, reduces opioid requirements and may lower pain scores up to 12 hours after cesarean delivery. In clinical practice, TAP block is usually combined with intrathecal morphine or fentanyl analgesics for intravenous analgesia. Various studies have indicated that ultrasound-guided bilateral TAP block can not only significantly reduce the frequency of maternal analgesia pump pressing, shorten the time of getting out of bed, and promote postpartum milk secretion but can also efficiently help reduce opioid dosage and opioid side effects.^[18–20]^ In 1 case report, TAP block catheters were used in 5 parturients and were found to prolong the time to the first opioid requirement and reduce pain scores.^[21]^

Few RCTs have investigated the use of TAP block catheters after cesarean delivery. Multiple meta-analyses have consistently reported,^[16,17,22]^ intrathecal morphine is preferable to TAP block for analgesia. Therefore, there was no further postoperative analgesic benefit with the TAP block when intrathecal morphine was administered.

In conclusion, we believe that the TAP block is a clinically feasible technique for postoperative analgesia following cesarean delivery, which can reduce opioid requirements and pain scores within 12 hours after cesarean section. This method not only improves the overall satisfaction of women who have given birth, but also makes postoperative analgesic treatment safer and more effective.

### 2.3. Ilioinguinal-iliohypogastric nerve block

The lumbar 1 spinal nerve root (L_1_) is the source of ilioinguinal (II) and iliohypogastric (IH) nerves. The IH nerve innervates the inguinal cutaneous area. However, the cutaneous sensations of the labia majora, scrotum, and medial thigh are innervated by nerve II, which passes through the inguinal canal.^[23]^ Therefore, the ilioinguinal-iliohypogastric (II-IH) nerve block provides anterior abdominal wall analgesia after cesarean section, especially in the region of the Pfannenstiel incision innervated by L_1_.^[24]^ II-IH nerve blocks produce somatic analgesia similar to TAP blocks, although they have less of an impact on visceral pain.

Studies have revealed that, for parturients not administered intrathecal morphine, a II-IH nerve block, provided by 1 or more injections, can offer useful analgesia following cesarean delivery.^[25,26]^ In a study by Bell et al,^[27]^ in which parturients received intraspinal anesthesia followed by a postoperative II-IH nerve block (which reported a 95% block success rate), they found a significant decrease in morphine use for maternal intravenous self-administered analgesia compared with the II-IH nerve block intervention group for 24 hours. However, the incidence of adverse reactions such as pain, pruritus, and nausea remained the same.^[27]^ Sakalli et al,^[25]^ who used Bell et al’s method of analgesia after cesarean delivery under general anesthesia, also showed a reduction in the dose of tramadol used in the II-IH nerve block group and a reduction in the rest pain score within 24 hours and the movement pain score within 8 hours after surgery. Still, no changes were observed in the occurrence of nausea, vomiting, or pruritus.

The II-IH nerve block also showed different results than the TAP block. Kiran et al showed a significant increase in the 24-hour average use of tramadol in the II-IH nerve block group compared to that in the TAP block group. However, there was no difference in the time to the first administration of intravenous analgesics and postoperative pain scores between the 2 groups.^[28]^ Conversely, in Ahemed et al’s^[29]^ nonrandomized prospective study, compared with TAP block after cesarean section, the 24-hour tramadol dose was lower in the II-IH nerve block group, and the time to first use of intravenous analgesics was also prolonged (14.09 hours vs 10.71 hours). At no time point, the pain scores of the 2 groups in the 2 studies differ significantly. However, we do not believe that the 2 studies can be directly compared because of the differences in the local anesthetic dose, drug delivery volume, and postoperative analgesia regimen. Staker et al’s study was distinctive in that it compared a control group receiving a noninvasive “mock block” to the combined II-IH nerve block and TAP block (I-TAP). In the initial 24 hours following surgery, the combination block group had decreased pain scores both during activity and at rest, as well as reduced opioid use at all times. The 2 groups did not differ in the occurrence of sedation, nausea, vomiting, or pruritus.^[30]^

In summary, II-IH nerve block can offer some analgesic benefits after cesarean delivery and reduce opioid consumption. It can also be used for cesarean delivery under general anesthesia or as an alternative remedial analgesic measure if other methods fail.

### 2.4. Rectus sheath block

The fibrous septum, known as the rectus sheath, is created by the aponeuroses of the transversus abdominis, internal oblique, and external oblique muscles that envelop the rectus abdominis. The thoracoabdominal nerve passes through the plane of the transversus abdominis muscle, enters the posterior sheath of the rectus abdominis muscle and branches into the midline of the abdominal wall. The arcuate line is the termination line of the posterior rectus abdominis sheath and the thoracoabdominal nerve runs anterior to the rectus abdominis muscle. If the conventional injection technique is used to inject drugs into the posterior layer of the rectus abdominis muscle, the nerves below the arcuate line may not be blocked and a consistent nerve block effect may not be produced.^[31]^

An RCT that compared patient-controlled intravenous morphine analgesia alone with a combination of rectus sheath block (RSB) for postoperative pain following cesarean delivery found no discernible variations in the ratings of postoperative pain and no decline in the 24-hour use of opioids.^[32]^ Therefore, RSB does not provide effective postoperative analgesia for cesarean delivery. The RCT also compared TAP block with RSB, demonstrating that the TAP block group had considerably lower postoperative pain scores and overall opioid use than the RSB group.^[32]^ One trial randomized 131 cesarean deliveries into intrathecal morphine with bupivacaine RSB, intrathecal morphine with saline RSB, or bupivacaine RSB alone. Compared with parturients who received intrathecal morphine with or without RSB, those who received only RSB required more analgesic medications over 48 hours. Intrathecal morphine decreased the postoperative pain scores at rest and during exercise, irrespective of whether the parturient received saline or bupivacaine RSB. Moreover, the rectus abdominis sheath block did not result in any further analgesic benefits of intrathecal morphine administration.^[33]^

Few studies have evaluated the effectiveness of low-Pfannenstiel incision cesarean sections for the treatment of postoperative pain in RSB. Further research is required to assess the analgesic effects of RSB.

### 2.5. Quadratus lumborum block

The target of the quadratus lumborum block (QLB) is the fascial plane surrounding the quadratus lumborum muscle, which is situated medial to the inferior border of the 12th rib and superior to the transverse processes of the L_1_–L_5_ vertebrae. The erector spinae is located behind the quadratus lumborum and the middle thoracolumbar fascia runs between them. The anterior thoracolumbar fascia lies between the quadratus lumborum and psoas major. The TAP is adjacent to the fascial plane and lateral to the quadratus lumborum. A lateral-approach QLB (QL-1) targets this boundary and allows diffusion of local anesthetics, similar to the TAP block. The posterior approach QLB (QL-2) targets the middle thoracolumbar fascia, and the anterior thoracolumbar fascia is the focus of the anterior approach QLB (QL-3).^[34]^ In intramuscular QLB in children, a local anesthetic is directly injected into the quadratus lumborum muscle.^[35]^ As QLB is associated with a greater posterior location than the TAP block, it is feasible for the local anesthetic to diffuse into the paravertebral space. Therefore, visceral and somatic pain analgesia may be covered by the QLB, and theoretically, when compared to the TAP block, the former may offer better analgesia.^[36]^

It has been shown that different QLB methods result in slightly different dermal spreads, with QL-1 and QL-2 approaches covering T_7_ to L_1_, QL-3 approach covering T_10_ to L_4_, and intramuscular QLB covering T_7_ to T_12_.^[37]^ RCTs have demonstrated that QL-1, QL-2, and QL-3 are beneficial in lowering parturients’ opioid use and postoperative pain scores following cesarean delivery compared with controls who were not administered intrathecal morphine.^[38–41]^ In addition, an RCT in 2021 compared the analgesic effects of QL-3 and QL-2 blocks in parturients undergoing intraspinal anesthesia without intrathecal morphine.^[42]^ In the QL-3 group, the scores for pain experienced after surgery and the number of opioid medications used within 24 hours decreased considerably. Furthermore, the duration was extended to the initial compression of the pain-relieving pump.^[42]^

Several meta-analyses have demonstrated that among parturients who did not receive intrathecal opioid postoperative analgesia, the QLB group experienced more significant analgesic effects than the blank control group. Nevertheless, in a meta-analysis that included ten RCTs, Tan et al^[43]^ discovered that when comparing intrathecal morphine alone with a simultaneous QLB, there was no discernible improvement in analgesic effect. The meta-analysis by El-Boghdadly et al^[44]^ on QL-1, QL-2, and QL-3 supports this conclusion. While the above analysis suggests that the QLB has limited efficacy in parturients already receiving intrathecal morphine, Salama et al showed that parturients receiving a single injection of 0.375% ropivacaine to block the quadratus lumborum muscle had significantly lower resting and motor pain scores than those receiving 0.1 mg intrathecal morphine. After 48 hours, the dosage of opioids was reduced and adverse reactions were significantly reduced.^[45]^ These results require further clinical verification.

In summary, our findings indicate that QLB can offer good postoperative pain relief in parturients for whom morphine administration is contraindicated, and does not provide additional benefits in those receiving intrathecal morphine.

Most of the studies mentioned above have focused on the acute analgesic effects of nerve blocks. Borys et al^[46]^ randomized women in labor who were not administered intrathecal opioids to receive a TAP block or QLB and assessed postoperative pain using the Neuropathic Pain Symptom Inventory several months later. Both groups showed significant reductions in pain scores compared to the control group (intravenous morphine self-administered analgesia only) at 1 and 6 months postoperatively. However, the study failed to demonstrate that the chronic pain scores significantly varied when comparing the QLB and TAP block groups.^[46]^ This particular study highlights that the advantages of regional blocks surpass the duration of the blocking drug, suggesting that future research should aim to expand and quantify this effect.

### 2.6. Erector spinae plane block

Erector spinae plane block (ESPB) is the result of administering a local anesthetic injection into the interfascial plane between the tips of the transverse processes of the vertebrae in the plane of the erector spinae.^[47]^ The effectiveness of ESPB in anterior chest wall surgery shows that the intercostal nerve is sometimes blocked, even though only the dorsal branch of the spinal nerve resides in this plane. Local anesthetics spread craniocaudally in this potential space and can theoretically spread anteriorly into the paravertebral space. Small cadavers and in vivo studies have shown that local anesthetics may spread anteriorly into the lumbar plexus.^[48]^ An investigation of the proliferation of local anesthetics in the ESPB showed that 3.4 mL a local anesthetic was required for distribution at the level of 1 dermatome, and that the local anesthetics eventually spread into the paravertebral space, acting on the anterior rami and roots of the spinal nerves.^[49]^ It is also possible for local anesthetics to migrate into the epidural area after entering the paraspinal or lumbar plexus region.^[50]^ Therefore, there is a possibility of epidural block following ESPB. In chest wall, abdomen, and spine surgeries, a common alternative to thoracic paravertebral block is ESPB, which is theoretically safer and more straightforward to execute.

ESPB is a comparatively recent technique in clinical use and has been used in recent years in studies on analgesia after cesarean section. Research has indicated that a bilateral ESPB at T_9_ can reduce the dose of fentanyl used 24 hours after surgery and prolong the time of the first analgesia pump in parturients who do not receive intrathecal morphine.^[51]^ Two RCTs compared the analgesic effects of ESPB and TAP blocks and revealed that the former was more effective and had a longer analgesic duration than the latter.^[52,53]^ A 2022 meta-analysis and systematic review that included 3 articles examining the effectiveness of ESPB for cesarean delivery showed that ESPB reduced total opioid use but did not reduce postoperative pain scores.^[54]^ An RCT revealed no discernible difference in pain levels or analgesic efficacy between posterior QLB and lower thoracic ESPB.^[55]^ Another RCT on anterior QLB and lower thoracic ESPB suggested that the 2 groups experienced the same analgesic effect.^[56]^ Most RCTs that evaluated ESPB after cesarean section did not use intrathecal opioids. However, in another RCT, Hamed et al^[57]^ evaluated the analgesic effectiveness of intrathecal morphine (0.1 mg) and ESPB, suggesting that the ESPB group had significantly decreased pain scores and oral opioid use in the first 24 hours, and that the first analgesic supplementation was prolonged.

In conclusion, relatively few studies have been conducted on the clinical application of ESPB for the treatment of postoperative pain after cesarean delivery. Further studies are required to confirm the safety and analgesic efficacy of these drugs.

### 2.7. Local wound infiltration analgesia

Wound infiltration analgesia involves injecting a local anesthetic directly into a specific location in the surgical area after surgery. However, there are no uniform standards for this technique. Local anesthetics can be injected deep into the fascia of the rectus abdominis muscle and into the plane between the subcutaneous tissue and the fascia of the rectus abdominis muscle. They can also be injected into the subcutaneous tissue at the incision site. In some cases, indwelling catheters can be inserted into the selected plane for continuous infusion of the local anesthetic.^[6]^ Studies suggest that a single wound-infiltration injection of a local anesthetic reduces intravenous morphine infusion by approximately 9 mg during the first 24 hours following cesarean delivery in those who are not receiving intrathecal morphine.^[58]^ Moreover, this does not minimize the adverse effects of opioids, which include nausea, vomiting, and pruritus.^[58]^ A meta-analysis that included 42 RCTs on TAP block and wound infiltration revealed no statistically significant differences between the 2 techniques with regard to the time to the first analgesic refill, 24-hour opioid use, or 24-hour pain scores.^[59]^ Another meta-analysis showed that TAP blocks had only a small or no significant advantage in lowering postoperative pain compared with local anesthetic wound infiltration analgesia.^[60]^

Local anesthetics and anti-inflammatory drugs such as diclofenac are commonly used for wound infiltration analgesia. A study by Lavand et al^[61]^ revealed that diclofenac wound infiltration decreased opioid use and pain scores during cesarean deliveries. Carvalho et al^[62]^ also discovered that following cesarean delivery, the analgesic dosage and pain scores decreased, and the interleukin-10 and interleukin-6 levels in the wound exudates decreased when ketorolac was added to local anesthetics. Similar research has demonstrated that the use of dexmedetomidine as an adjuvant enhances analgesia compared to the use of local anesthetics alone.^[63]^

Single-wound infiltration analgesia when intrathecal morphine is administered is of no additional benefit to parturients undergoing cesarean delivery but may be beneficial to those requiring general anesthesia or for whom intrathecal morphine is contraindicated.^[58]^ In 1 study that compared intrathecal morphine analgesia with continuous wound infiltration analgesia, intrathecal morphine provided better analgesia, reduced opioid consumption within 12 hours after surgery, and extended the period before the need for analgesics.^[64]^ Studies on persistent wound infiltration have repeatedly reported increased wound fluid leakage and complications, such as wound hematoma and wound infection, requiring repeated dressing changes and affecting maternal satisfaction.^[65,66]^ Therefore, the application of wound infiltration analgesia in postoperative analgesia after cesarean delivery is limited and can only be used as an auxiliary analgesic method.

### 2.8. Intraperitoneal instillation of local anesthetics

Intraperitoneal instillation of local anesthetic is a quick and simple technique that has been poorly studied for the treatment of pain after cesarean delivery. Werntz et al^[67]^ reported intraoperative pain relief after cesarean delivery using a 3% chloroprocaine intraperitoneal drip for cesarean delivery under intraspinal anesthesia. Patel et al^[2]^ found that intraperitoneal infusion of 20 mL of a mixture of epinephrine and 2% lidocaine decreased pain scores 2 hours after surgery but had no effect on pain scores 24 hours after surgery or the amount of opioids used after surgery. The subgroup analysis indicated that intraperitoneal instillation of 20 mL of a mixture of epinephrine and 2% lidocaine reduced pain scores during 24-hour exercise in parturients with peritoneal closure, but not in those without peritoneal closure.^[2]^ In another study involving parturients undergoing cesarean delivery who underwent closure of the mural peritoneum, intraperitoneal instillation of local anesthetics decreased opioid use, opioid-related adverse events, and postoperative pain levels compared to placebo.^[68]^ The pharmacokinetics of local anesthetics following intraperitoneal instillation has not been extensively studied, and further clinical research is required to establish the ideal dosage of local anesthetics to balance safety and efficacy. Intraperitoneal spraying of local anesthetics has not been adequately studied as a technique to reduce pain following cesarean delivery. This method may be beneficial for parturients undergoing peritoneal sealing; however, further trials are needed to verify this.^[69]^ When intrathecal morphine analgesia is not administered, the application of intraperitoneal local anesthetics should be compared with other regional block techniques to guide clinical selection.

## 3. Conclusion

Adequate analgesia after cesarean section can promote the health of mothers and infants, and has a significant impact on the postoperative rehabilitation and quality of life of parturients. There are many methods of postoperative analgesia after cesarean section. With the expanding concept of fast-track surgery, regional nerve blocks have become popular postoperative analgesics. In summary, intrathecal morphine analgesia remains the gold standard postoperative analgesia after cesarean delivery. Regardless of the regional nerve block approach used, the short-term advantages of a single regional block may not be significant in parturients receiving intrathecal morphine. Regional blocks have been shown to enhance postoperative analgesia and reduce postoperative opioid intake in parturients unable to receive intrathecal morphine. Studies have indicated that the analgesic effect appears to be more significant when the regional block is closer to the sympathetic trunk or the spinal nerve root. More studies support the application of the TAP block than ESPB; however, we believe that ESPB may have more clinical advantages. Further clinical studies are required to verify this hypothesis.

## Acknowledgments

We would like to thank Editage (www.editage.cn) for the English language editing. No external funding was received for the study.

## Author contributions

**Conceptualization:** Sulan Qin.

**Formal analysis:** Yujiao Yang, Sulan Qin, Yongyi Qin, Zhaohui Xiong.

**Project administration:** Sulan Qin, Zhaohui Xiong.

**Writing – original draft:** Yongyi Qin.

**Writing – review & editing:** Yujiao Yang, Sulan Qin.
